# Seeking Membranes: Positive-Strand RNA Virus Replication Complexes

**DOI:** 10.1371/journal.pbio.0060270

**Published:** 2008-10-28

**Authors:** Mark R Denison

## Abstract

How much do we really understand about how +RNA viruses usurp and transform the intracellular architecture of host cells when they replicate?

Viruses containing RNA genomes, or RNA viruses, constitute an extensive genetic group of plant, animal, and human viruses. RNA viruses include many viruses that are important human pathogens, and have been shown to have the ability to move from animals to humans, resulting in zoonotic-human epidemics. RNA viruses that contain single-stranded RNA genomes of positive polarity are known as positive-strand RNA (+RNA) viruses. +RNA virus families of animals and humans include the Coronaviridae (SARS-coronavirus), Picornaviridae (poliovirus, hepatitis A), Flaviviridae (dengue, West Nile virus) and Togaviridae (Venezuelan equine encephalitis virus, rubella virus). The objective of this Primer is to focus on established and new approaches to understand the interface of +RNA virus replication and cell biology.

## The Complexities of +RNA Virus Cell Biology

Positive-strand viruses replicate in the cytoplasm of the host cell in association with cytoplasmic membranes. To complete a productive infection, they must enter cells, institute cytoplasmic replication factories, replicate genome RNA, express their genomes, sort and traffic proteins and RNA, and assemble and release virus particles—in some cases within an 8–12 hour life cycle. Investigators who seek to dissect the replication strategies of +RNA viruses are drawn inexorably into the complexities of cellular biology. To determine the mechanisms by which +RNA viruses navigate these steps may necessitate an understanding of cell biology processes as diverse as cytoplasmic organelle structure and membrane biogenesis, endoplasmic reticulum (ER) formation, microtubule motor movement, actin polymerization, and trafficking in the secretory pathway. +RNA viruses also share a common requirement for translation of the input mRNA-sense +RNA genome to yield nonstructural proteins that are responsible for both induction of membrane modifications and RNA replication and transcription. These genomes range in size and complexity from the small, segmented 4.5 kb flock house virus (FHV) genome to the 27–32 kb nonsegmented coronavirus (CoV) genomes, which express an ~800 kDa replicase fusion polyprotein processed by multiple viral proteinases into 16 mature nonstructural proteins (nsp1 to 16) [1].

## Co-Opting Host Membranes during +RNA Virus Replication

All studied +RNA viruses usurp and modify cytoplasmic membranes for formation of functional sites of protein translation, processing, and RNA synthesis, as described in detail in recent reviews [2–5]. These sites, containing cellular membranes, viral RNA, and viral replicase proteins, are generally referred to as replication complexes (RCs). This term is an accepted working imprecision, especially in comparison with the more defined protein/DNA/RNA RCs of cellular replication. RCs of +RNA viruses are more akin to factories than molecular machines, since they mediate or coordinate multiple functions at different sites during the course of infection. Specifically, +RNA genomes must function in RCs as templates for translation, minus-strand synthesis, and packaging into progeny virions, as well as for amplifying RCs over the course of infection. In addition, RCs also likely contain multiple cellular proteins, although for most +RNA viruses these remain to be defined by proteomic analysis of purified RCs.

## New Tools, Better Models

There are many critical unanswered questions in the cell biology of +RNA virus RC formation and function: How are the virus-induced membrane modifications regulated to optimize their formation and functioning in viral RNA synthesis? How are membrane-associated complexes organized to achieve the seemingly opposed requirements of protecting replicating RNA while maintaining access to proteins, nucleotides, and the maturation and assembly of new progeny virions? How do +RNA viruses direct and regulate the movement of new genome RNA from RCs to sites of assembly that may be associated with distinct membranes or organelles? Do viruses have absolute requirements for specific membrane types that are determinative in cell selection and tropism? How do viruses sort new genome for RC amplification, replication, and assembly?

For many +RNA viruses, studies using visible, immunofluorescence, and electron microscopic imaging have begun to address these questions by demonstrating association of viral replicase proteins and RNA with cellular membranes derived from a variety of organelles, including ER, late-endosomes/lysosomes, and mitochondrial outer membranes (Table 1) [3]. More recently, high-pressure cryofixation combined with electron microscopy (EM), immuno-EM, electron microscopic tomography, 3-D reconstruction, and direct correlation of physical and biochemical analysis of RCs have yielded much more defined and testable models of RCs. The viral RCs are made up largely of viral proteins and enzymes about which we know very little. Researchers have developed their visualization systems as a way to study these proteins and to work out how they interplay with the modified host membranes to ensure viral replication and spread.

**Table 1:**
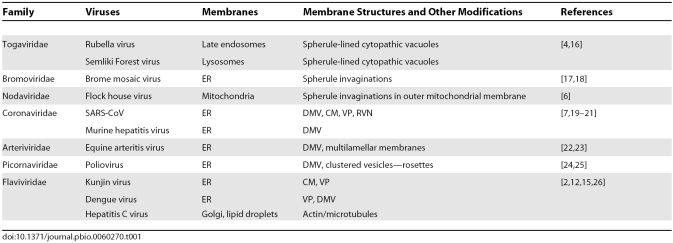
Membranes Modifications Induced by Positive-Strand RNA Viruses

An example of this is the nodavirus FHV, which forms discrete spherular invaginations in outer mitochondrial membrane [6]. The spherules appear to obey relative rules of size and protein and RNA content consistent with predictions based on immuno-EM and biophysical measurements. In addition, the spherules have open necks to the cell cytosol predicted to be sufficient for movement of RNA molecules. The power in this study is derived in part from the fact that FHV expresses the single multifunctional Protein A, which is responsible for both membrane modifications and viral RNA synthesis and thus can be directly correlated with the immunofluorescence and ultrastructural studies.

## Illuminating Replication Strategies Used by SARS-CoV

The present study in *PLoS Biology* by Knoops et al. [7] uses similar tools to analyze the membrane modifications that occur during SARS-CoV infection of Vero cells. The authors developed a sophisticated method to preserve and visualize the fragile replication structures of SARS-CoV, both in whole cells by light microscopy and in sections of cells by electron microscopy. In contrast to FHV, the coronaviruses, as modeled by SARS-CoV, express a replicase polyprotein that gives rise to up to 16 mature nsps that colocalize in cytoplasmic foci. These include three integral membrane proteins or nsps that likely mediate membrane association and modification (nsp3, 4, and 6), and at least six nsps proven or predicted to participate in RNA synthesis and maturation (nsp 8, 12–16). In addition, the membrane origins, modifications, and evolution of CoV RCs appear to have been more complex and varied based on reports with different CoVs in different cell lines. This study describes in SARS-CoV-infected Vero cells a reticulovesicular network (RVN) with closely associated, interdigitated, and connected components of double-membrane vesicles (DMVs), convoluted membranes (CMs), vesicle packets (VPs), and budding virions with possible differential localization of replicase proteins, viral double-stranded RNA, and virion budding regions.

The study is important for the CoV and +RNA virus cell biology field for several reasons. First, it suggests more common mechanisms between coronaviruses and other +RNA viruses, such as the Flaviviridae, that also induce DMVs, CMs, and VPs. Second, it may allow the unification of seemingly disparate results of previous studies of CoV RC membrane origins and evolution. Third, it presents a model for testing questions that have been raised by other studies. For example, sites of CoV virion assembly appear to be distinct from RCs, but it is not known how progeny genome RNA is delivered from RCs to sites of assembly. Analysis of the RVN over time with multiple cellular and replicase markers will likely define the mechanism for RNA and protein movement from RCs to sites of assembly. Finally, the model raises exciting new questions. The results suggest that the majority of SARS-CoV double-stranded RNA, and possibly RNA synthesis, resides within the interior of the DMV vesicles, while virion budding occurs from the cytosol. However, the careful 3-D reconstructions did not identify any vesicle opening or pore in the DMVs that would allow movement of genome RNA from the inside to the cytosol. This suggests a novel mechanism both for access to the membrane-protected interior and for egress of genome RNA.

## Whither +RNA Virus Cell Biology?

The integration of novel approaches for high-resolution reconstruction of +RNA RCs is an exciting evolution of viral cell biology. Future studies to address the remaining and new questions of RC formation and function will require approaches to define the RCs by their viral and cellular proteomes, and to determine protein interactions, membrane changes, RNA synthesis, and virion assembly. Recent studies have tracked viral RNA in real time by tagging it with green fluorescent proteins (GFPs) or “molecular beacons.” Engineered viruses expressing GFP-fusions with viral replicase proteins have been used for direct fluorescent studies of replicase protein targeting, and will allow real-time analysis of protein expression, interactions, and movement [8]. GFP-replicase protein fusions have also been used for immunoisolation and proteome analysis of RC-associated viral and cellular proteins using high-affinity anti-GFP antibodies [9]. Approaches exist for direct FACS (fluorescence activated cell sorting)-assisted vesicle sorting based on dual fluorescence of membrane integral dyes and GFP-fusion proteins, which may allow a complementary approach to proteome analysis that does not require affinity approaches and may allow analysis of intact RCs [10]. GFP-replicase fusion proteins and insertion of tetracysteine tags also will facilitate correlation of live-cell fluorescence and EM analysis of replicase proteins [11]. Finally, it will likely be necessary to track +RNA viruses through new cellular landscapes. For example, lipid droplets are involved in hepatitis C virus RCs [12]. Microtubules and microtubule organizing centers have been implicated in formation and functions of pericentriolar sites of virus assembly and replication [13]. Requirements for delivery of genome RNA from RCs to sites of virion assembly may also implicate association and possible functions of microtubule-mediated vesicle movement as reported for hepatitis C virus [14,15].

In summary, the level of resolution and understanding of +RNA RC formation and function is rapidly increasing, and new technologies are being developed and applied to understand how “simple” +RNA viruses can usurp and transform the intracellular architecture in a rapid and comprehensive manner. We will undoubtedly continue to be surprised by the approaches used by the viruses and the insights they provide into the fundamental biology of the cell.

## Abbreviations

CM: convoluted membrane
CoV: coronavirus
DMV: double-membrane vesicle
EM: electron microscopy
ER: endoplasmic reticulum
FHV: flock house virus
GFP: green fluorescent protein
nsp: nonstructural protein
RC: replication complex
+RNA: positive-strand RNA
RVN: reticulovesicular network
VP: vesicle packet
